# Hydrogen Embrittlement and Improved Resistance of Al Addition in Twinning-Induced Plasticity Steel: First-Principles Study

**DOI:** 10.3390/ma12081341

**Published:** 2019-04-24

**Authors:** Lilin Lu, Jiaqi Ni, Zhixian Peng, Haijun Zhang, Jing Liu

**Affiliations:** 1 State Key Laboratory of Refractories and Metallurgy, School of Materials and Metallurgy, Wuhan University of Science and Technology, Wuhan 430081, China; jiaqini_wust@163.com (J.N.); wh_pzx@163.com (Z.P.); zhanghaijun@wust.edu.cn (H.Z.); 2 Hubei Province Key Laboratory of Coal Conversion and New Carbon Materials, School of Chemistry and Chemical Engineering, Wuhan University of Science and Technology, Wuhan 430081, China

**Keywords:** hydrogen embrittlement, austenite, Al addition, elastic constants, first-principles study

## Abstract

Understanding the mechanism of hydrogen embrittlement (HE) of austenitic steels and developing an effective strategy to improve resistance to HE are of great concern but challenging. In this work, first-principles studies were performed to investigate the HE mechanism and the improved resistance of Al-containing austenite to HE. Our results demonstrate that interstitial hydrogen atoms have different site preferences in Al-free and Al-containing austenites. The calculated binding energies and diffusion barriers of interstitial hydrogen atoms in Al-containing austenite are remarkably higher than those in Al-free austenite, indicating that the presence of Al is more favorable for reducing hydrogen mobility. In Al-free austenite, interstitial hydrogen atoms caused a remarkable increase in lattice compressive stress and a distinct decrease in bulk, shear, and Young’s moduli. Whereas in Al-containing austenite, the lattice compressive stress and the mechanical deterioration induced by interstitial hydrogen atoms were effectively suppressed.

## 1. Introduction

Hydrogen embrittlement (HE) effect on the mechanical properties of metallic alloys has attracted enormous research attention and has become a popular issue in industrial applications [1,2,3,4,5]. It has been reported that a large variety of damages to the metallic materials are caused by HE, including stress corrosion cracking and H-induced delayed cracking. These usually lead to a significant reduction of the mechanical properties of alloy materials [6,7,8]. Twinning-induced plasticity (TWIP) steel, which has typical compositions of austenite, is currently one of the most attractive materials for extensive applications in the automotive industry. Due to the deformation twinning of face-centered cubic (FCC) structure [3], TWIP steel has ultimate strength (>1000 MPa) and superior tensile elongation (elongation to failure of >50%) [9]. However, significant deterioration in its elongation and ductility can be induced by hydrogen-charging experiments. In situ electron channeling contrast imaging of Fe-Mn-C TWIP steel has revealed that hydrogen-assisted cracking initiates at both grain boundaries and deformation twins, the stress concentration at the tip of deformation twins plays an important role in HE [10]. 

Several attempts have been made to elucidate the mechanism of deterioration of mechanical properties caused by HE. H-enhanced decohesion (HEDE) [11] and H-enhanced local plasticity (HELP) [12,13,14] are two popular mechanisms concerning HE, but many contradictory observations indicate the uncertainty of them [15,16]. Based on a large-scale molecular dynamics study, hydrogen accumulation at the crack tip is deemed to be the reason for the transition from ductile to brittle, and a hydrogen-triggered ductile-to-brittle transition mechanism has been proposed by Curtin et al. [17]. 

To reduce the unfavorable effect caused by HE, many strategies such as adjusting grain size, forming alloy protective coating and addition of some metallic elements have been developed [18,19,20,21,22,23]. Among them, Al addition was proved to be a promising strategy of improving the resistance of TWIP steel to HE [24,25,26], and understanding of the mechanism underpinning it has attracted great research interests. Song et al. have found that the resistance of TWIP steels to HE can be effectively improved by Al addition at high strain amplitude via preventing intergranular cracking, whereas at low-strain amplitude the initiation of fatigue cracks was promoted by Al addition [27]. In other work, the possible reason for preventing intergranular cracking was described as the low hydrogen-absorption ability of Al-containing TWIP steels [28,29], but thermal desorption spectroscopy analysis yielded contradictory conclusions, which suggested that the amount of hydrogen absorbed in Al-containing steel was greater than that in Al-free steel [30]. A first-principles calculation revealed that Al atoms could cause a localized dilation in TWIP steel, this is favorable for hydrogen absorption and trapping, which limits hydrogen diffusion in TWIP steel. [31]. 

In this work, first-principles studies have been performed to explore the HE mechanism and the improved resistance of Al-containing austenite to HE. Firstly, the structures of interstitial compounds between hydrogen atoms and austenite, with a special focus on interstitial site preference of hydrogen atoms, have been investigated. Secondly, the effects of interstitial hydrogen on lattice compressive stress and elastic constants of austenitic steel were investigated. Finally, the HE mechanism of austenitic steel and the relationship between improved resistance to HE and Al addition were discussed. 

## 2. Computational Methods

**Figure 1:**
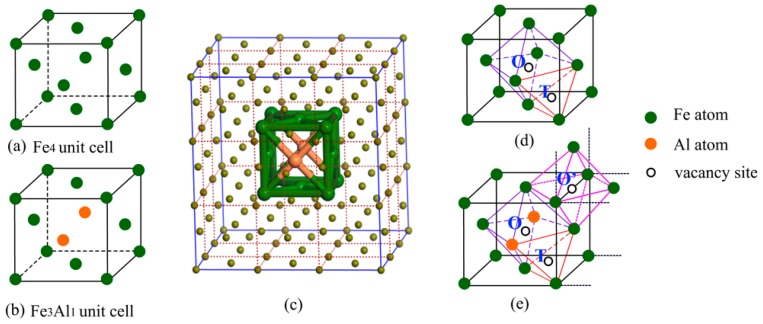
The face-centered cubic (FCC) unit cell of Al-free austenite Fe_4_ (**a**), Al-containing austenite Fe_3_Al_1_ (**b**), 3 × 3 × 3 supercell of FCC structures for Al-containing austenite (**c**), the interstitial sites in Al-free austenite (**d**) and Al-containing austenite (**e**).

In this work, TWIP steel was simulated by using face-centered cubic (FCC) structure since it has typical compositions of austenite [5,31], and Fe_4_ and Fe_3_Al_1_ unit cells were selected to study interstitial site preference of hydrogen atoms in Al-free TWIP steel and Al-containing TWIP steel, respectively (Figure 1a,b). First-principles calculations were implemented using the Cambridge serial total energy package (CASTEP), density functional theory (DFT) [32] within generalized gradient approximation (GGA) of the Perdew–Burke–Ernzerhof (PBE) functional [33] was used to consider electron exchange and correlation. Interactions between the core region and valence electrons were described using the ultrasoft pseudopotentials (USP) [34] with a plane wave basis set cutoff energy of 400 eV. The equilibrium geometries were determined by performing optimization with cell parameters and atomic coordinates fully relaxed using the Quasi-Newton method with a Broyden–Fletcher–Goldfarb–Shannon (BFGS) update of the Hessian [35], a 14 × 14 × 14 *k*-point Γ-centered Monkhorst-Pack grid was selected to sample the Brillouin zone. The complete linear synchronous transit/quadratic synchronous transit (LST/QST) method [36] was employed to search the transition states (TS), which were confirmed by the nudged elastic band method [37]. Furthermore, a 3 × 3 × 3 supercell of FCC structures for Al-containing austenite (Fe106Al2, Figure 1c), in which the theoretical Al content (1.8 wt.%) approaches the experimental value (less than 2 wt.%), was selected to simulate the Al-containing TWIP steel materials in actual situation to calculate the elastic constants, and a 3 × 3 × 3 supercell of Al-free austenite (Fe108) was also studied for comparison.

Many magnetic states have been used to simulate austenite in previous works. Nonmagnetic (NM) state was applied to investigate the interaction between aluminum and hydrogen in TWIP steel and the effect of carbon on the stacking fault energy of Fe–C alloys [31,38]. The paramagnetic state was used to study the vacancy formation energy in iron, and excellent agreement was obtained between theory and experiment [39]. Antiferromagnetic double layer (AFMD) structure gives an agreeable result of thermal expansion with experimental findings, but increasing temperature deteriorates the agreement [40]. Generally, FCC Fe is experimentally found to be paramagnetic at ambient temperature, but the nonmagnetic (NM) phase such as ferrite magnetic low spin (FM–LS) phase often degenerates with the paramagnetic phase [41]. A comparative study showed only a small energy difference between ferromagnetic (FM) state and NM state [42]. Since the structures in different magnetic states are very close in energy, the maximum energy difference is only 0.062 eV/atom [43], NM and FM states are both taken into account when we performed first-principles studies in this work. 

To investigate the site preference of interstitial hydrogen atoms in Al-free and Al-containing austenites, the binding energies (Δ*H*_bind_) were calculated as the energy difference between the sum of austenite and hydrogen atoms and the interstitial compound according to the following equation, Δ*H*_bind_ = *H*(austenite) + n*H(H-atom) – *H*(interstitial), where *H*(austenite), *H*(H-atom), and *H*(interstitial) are the total energies of the primitive cell of austenite, H-atom, and interstitial compound, respectively. *n* is the number of interstitial hydrogen atoms included in the austenite unit cell. The mean compressive stress at the unit cell surface, which is derived from the potential components of the virial theorem, was calculated to explore the influence of interstitial hydrogen atom on the lattice compressive stress in Al-free and Al-containing austenites. 

The theoretical elastic constants were calculated from the energy variation by applying small strains to the equilibrium configurations of Al-free and Al-containing supercells in NM states according to the method described in published work [44]. The bulk modulus (*B*) and shear modulus (*G*) have been derived from the three independent elastic constants, *C*_11_, *C*_12,_ and *C*_44_ according to the formulas [45],

*B*_V_ = *B*_R_ = (*C*_11_ + 2*C*_12_)/3

*G*_V_ = (*C*_11_ − *C*_12_ + 3*C*_44_)/5, *G*_R_ = 5(*C*_11_ − *C*_12_)*C*_44_/[4*C*_44_ + 3(*C*_11_ − *C*_12_)]

*B* = (*B*_V_ + *B*_R_)/2, *G* = (*G*_V_ + *G*_R_)/2

The Young’s modulus *E* was obtained by the following formulas [45,46].

*E* = 9*BG*/(3*B* + *G*)

*B*_V_, *B*_R_, *G*_V,_ and *G*_R_ are the Voigt bulk modulus, Reuss bulk modulus, Voigt shear modulus and Reuss shear modulus, respectively. 

## 3. Results and Discussion

### 3.1. Sites Preference of Interstitial Hydrogen Atoms in Austenite and Al-Containing Austenite

The lattice parameter for NM austenite was calculated to be 3.45 Å, which is consistent with the reported value (3.44–3.45 Å) [31,40]. While for FM austenite, the calculated lattice parameter was 3.91 Å, slightly more than the calculated 3.64 Å and the experimentally observed 3.65 Å. For Al-containing austenite, the FCC lattice undergoes a somewhat orthorhombic distortion in both NM and FM states.

Two kinds of interstitial sites, i.e., octahedral sites (O site) and tetrahedral sites (T site) (displayed in Figure 1d), were reported to be occupied by hydrogen atoms in austenite [47]. Here, the site preference of interstitial hydrogen atoms in Al-free and Al-containing austenites has been investigated based on the total energies of interstitial compounds (Table 1). In NM state, hydrogen atom prefers to occupy the octahedral site (O site), and the interstitial compound with hydrogen atoms at the O interstitial site is energetically lower than that with the hydrogen atoms at the T interstitial site by 0.38 eV. The situation in FM state is opposite to that in NM state, the interstitial compound with hydrogen atom occupying the T interstitial site is more stable, with the energy difference of only 0.06 eV. This shows that the magnetic state distinctly affects the priority of the occupational site of the interstitial hydrogen atoms in the Al-free austenite.

In Al-containing austenite, hydrogen atoms probably occupy the three interstitial sites, i.e., the tetrahedral site (T site) and two octahedral sites differentiated by ligand atoms (O and O’ site) (Figure 1e). Our results demonstrate that the interstitial compound with the hydrogen atoms at the T interstitial site is unstable in NM state, relaxation of this structure leads to hydrogen atoms ultimately occupying the O’ interstitial site, thus forming a more stable interstitial compound as compared to that with the hydrogen atoms at the O interstitial site. The energy difference between these two interstitial compounds is approximately 0.83 eV. In FM state, hydrogen atoms can occupy T, O, and O’ sites. By comparison, the configuration with hydrogen at the O’ interstitial site is the most stable; this is the same as the case in NM state, indicating that the magnetic state shows negligible influence on the site preference of interstitial hydrogen in Al-containing austenite.

**Figure 2:**
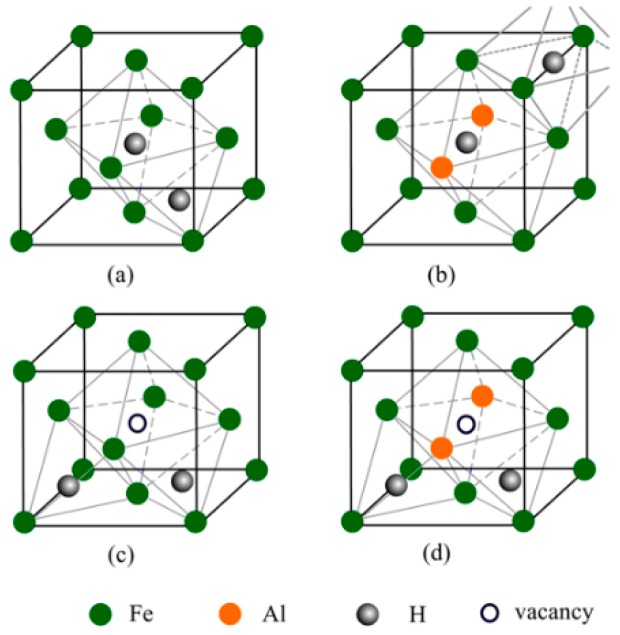
The atomic structures of the most stable interstitial compounds of Al-free and Al-containing austenites including two hydrogen atoms in NM state (**a**,**b**) and FM state (**c**,**d**).

**Figure A1:**
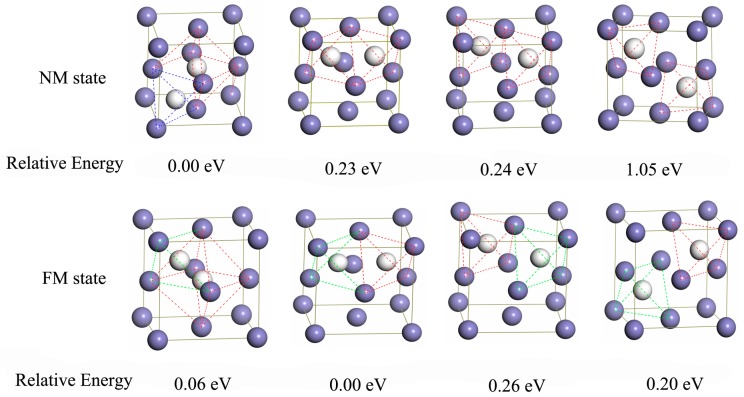
The structures of interstitial compounds of Al-free austenite containing two hydrogen atoms and the relative energy (eV) in NM and FM states.

**Figure A2:**
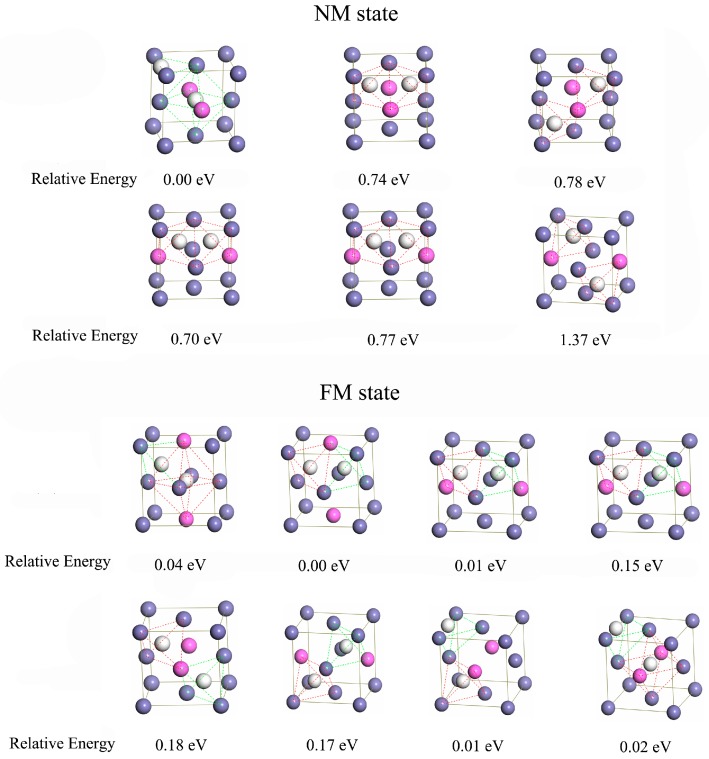
The structures of interstitial compounds of Al-containing austenite containing two hydrogen atoms and the relative energy (eV) in NM and FM states.

The interstitial compounds including two hydrogen atoms were also investigated, and a wide variety of configurations were displayed in Figure A1 and Figure A2 (in Appendix A). In NM state, the interstitial compound with the hydrogen atoms at O and T interstitial sites is the most stable for austenite (Figure 2a). For the Al-containing austenite, the configuration with two hydrogen atoms at the octahedral sites (O and O’ site) is the most stable (Figure 2b). In FM state, the most stable configurations are that with two hydrogen atoms at tetrahedral sites (T site) for both Al-free and Al-containing austenites (Figure 2c,d). 

### 3.2. Binding Energies and Diffusion Barrier of Interstitial Hydrogen Atom in Al-Free and Al-Containing Austenites

The binding energies between austenite and interstitial hydrogen atoms have been calculated to understand the effect of Al addition on the mobility of interstitial hydrogen atoms (Table 1). In NM states, the binding energy of 3.24 eV in Al-free austenite (preferential O site) is remarkably lower than that of 3.85 eV in Al-containing austenite (preferential O’ site). The binding energy with two hydrogen atoms in Al-free austenite is calculated to be 5.48 eV, which is also significantly lower than that in Al-containing austenite (6.81 eV). In FM state, the binding energies between one hydrogen atom with Al-free and Al-containing austenites are 2.44 and 2.99 eV, respectively. When two hydrogen atoms are included, the corresponding binding energies are 5.36 and 5.50 eV, respectively. The higher binding energies indicate that hydrogen atoms can be fixed more stably by Al-containing austenite.

**Figure 3:**
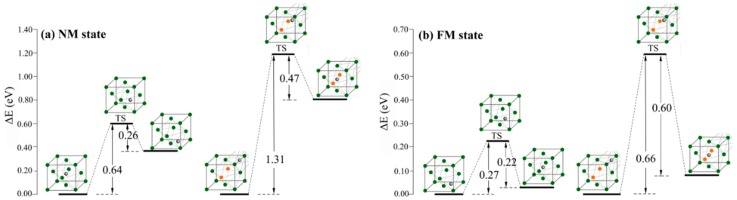
The calculated energetic profiles for interstitial hydrogen diffusion process in Al-free and Al-containing austenites in NM state (**a**) and FM state (**b**).

Doping heteroatoms is a promising strategy to fabricate the hydrogen trap for controlling hydrogen diffusion in metallic material. Herein, the interstitial hydrogen diffusion barriers in Al-free and Al-containing austenites were calculated to investigate the effect of Al atoms on the interstitial hydrogen diffusion capability. Figure 3 displays the energetic profiles of the hydrogen diffusion process. In the NM state, the transfer of interstitial hydrogen from the most preferential octahedral site to the tetrahedral site has the barrier height of 0.64 eV for Al-free austenite. For Al-containing austenite, the barrier height of hydrogen atom transfer from the most preferential O’ interstitial site to the O interstitial site is 1.31 eV. In FM state, the barrier of hydrogen transfer in Al-free austenite from the most preferential T interstitial site to the O interstitial site is 0.27 eV. In Al-containing austenite, the hydrogen transfer from O’ interstitial site to O interstitial site has the barrier height of 0.66 eV. The diffusion barrier in Al-containing austenite is distinctly higher than that in Al-free austenite indicating that Al atoms play a very important role in decreasing hydrogen mobility; Al-containing austenite is a better trap for the interstitial hydrogen atoms. This result is consistent with that reported in previous works [31].

### 3.3. Lattice Compressive Stress of Austenite and Al-Containing Austenite Caused by Osmotic Hydrogen

Previous works concerning HE mainly emphasized the effect of hydrogen on crack, dislocation, or other defects in steel, but little attention has been paid to the lattice stress induced by interstitial hydrogen atoms, which might be the origin of the remarkable reduction of mechanical properties of iron materials. 

As shown in Table 2, the interstitial hydrogen atoms have caused a distinct increase in lattice vectors and volumes of Fe_4_ and Fe_3_Al_1_ unit cells. However, the lattice vectors and volumes negligibly increase with existence of interstitial hydrogen atom when the Fe_4_ and Fe_3_Al_1_ unit cells are placed at the center of a 3 × 3 × 3 supercell (labeled as Fe_4_@Fe_108_ and Fe_3_Al_1_@Fe_106_Al_2_ in Table 2), indicating that unit cell expansion induced by interstitial hydrogen atom has been remarkably confined by the surrounding unit cells. 

**Figure 4:**
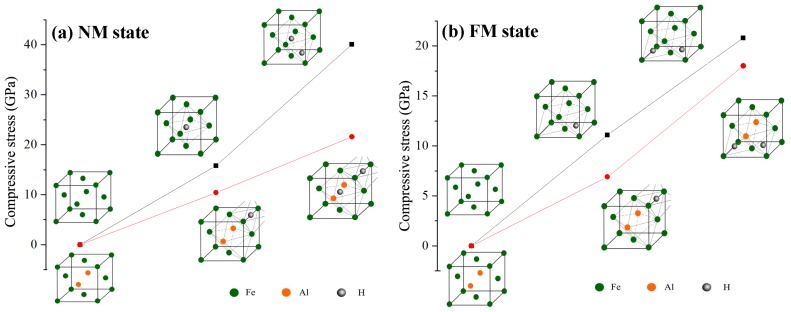
Schematic representation of the change in compressive stress of Al-free and Al-containing austenites in the NM state (**a**) and the FM state (**b**).

Figure 4 shows the lattice compressive stress induced by interstitial hydrogen atoms in Al-free and Al-containing austenites. In NM state, the compressive stress increases from approximately 0.0 GPa in Al-free austenite to 15.8 GPa with one hydrogen atom occupying the octahedral interstitial site and then increases to 40.1 GPa with two interstitial hydrogen atoms respectively occupying octahedral and tetrahedral interstitial sites. For Al-containing austenite, the compressive stress increases from approximately 0.0 GPa to 10.4 GPa with one interstitial hydrogen atom being included. When two hydrogen atoms are included, the compressive stress increases to 21.6 GPa. A similar trend is also presented in FM state, with one hydrogen atom occupying interstitial site in Al-free austenite, the compressive stress increases from 0.0 GPa to 11.1 GPa and then increases to 20.8 GPa for the case of two hydrogen atoms occupying interstitial sites. For Al-containing austenite, the compressive stress increases from 0.0 GPa to 6.9 GPa and 18.0 GPa, corresponding to one and two interstitial hydrogen atoms contained in the unit cell, respectively. As can be concluded from Figure 4, more interstitial hydrogen atoms resulted in a greater increase in compressive stress. Both in NM and in the FM state, Al atoms suppressed the increase in lattice compressive stress caused by interstitial hydrogen atoms, even though the effect of Al in FM state is not as much as that in NM state when more than one hydrogen atom exist at the interstitial site.

### 3.4. Elastic Constants and Mechanical Properties

**Figure 5:**
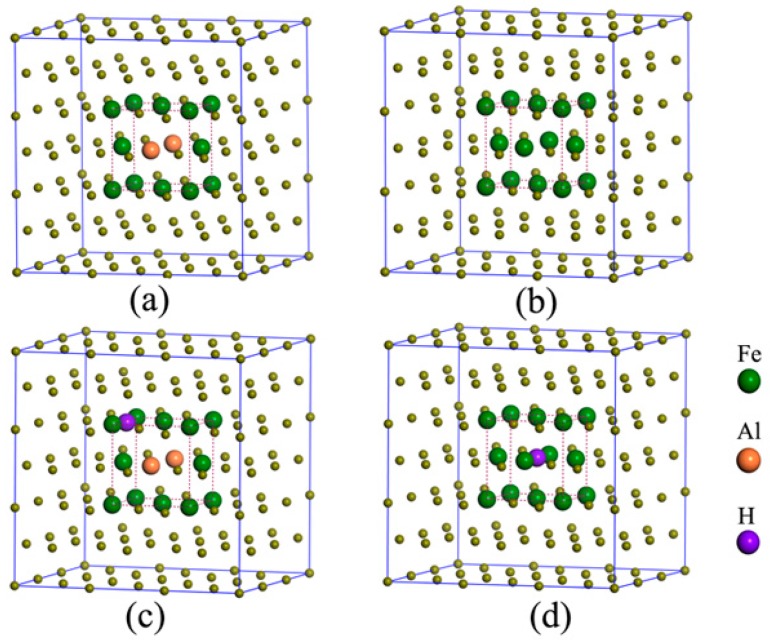
The atomic structure of Fe_106_Al_2_ (**a**), Fe_108_ (**b**), Fe_106_Al_2_H (**c**), and Fe_108_H (**d**).

Resonant ultrasound spectroscopic techniques, nanoindentation experiment and ab initio calculations are the common pathways usually used to determine the elastic constants of solid such as TWIP steel [48,49,50]. Herein, a supercell (3 × 3 × 3) with Al-containing unit cell at the center [Fe_106_Al_2_, approximately 1.8 wt.% Al, Figure 5a,c] was selected to perform the first-principles calculations to investigate the elastic constants in NM state, and a supercell (3 × 3 × 3) of pure Fe austenite [Fe_108_, Figure 5b,d] was studied for comparison.

The calculated elastic constants *C_ij_*, bulk modulus *B*, shear modulus *G* and Young’s modulus *E* of Al-free and Al-containing austenites were listed in Table 3. For a stable cubic structure, three independent elastic constants, i.e., *C*_11_, *C*_12_, and *C*_44_, should satisfy the Born stability criteria: *C*_11_ > *C*_12_, *C*_44_ > 0, and *C*_11_ + 2*C*_12_ > 0. Table 3 shows that the calculated elastic constants of Al-free austenite and Al-containing austenite both satisfy the Born stability criteria regardless of whether interstitial hydrogen is included or not, indicating that these investigated structures in NM state are all mechanically stable. The calculated *C*_11_, *C*_12_, and *C*_44_ of Fe_108_ are 473.9, 230.1 and 285.6 GPa, respectively. These values are in good agreement with the elastic constants (*C*_11_, *C*_12_, and *C*_44_ were 484, 234 and 287 GPa, respectively) reported in theoretical work [51]. 

Bulk modulus *B*, shear modulus *G*, and Young’s modulus *E* (in GPa) were also calculated to study the influence of interstitial hydrogen atom (Table 3). The bulk, shear, and Young’s moduli of Al-free austenite were 311.3, 203.0, and 500.2 GPa, respectively. The calculated shear modulus *G*_V_ and *G*_R_ of Al-free austenite are 220.1 and 185.8 GPa, respectively. These are very close to the reported values in Asker’s work [52]. When hydrogen atoms are included, the bulk, shear, and Young’s moduli decrease to 301.2, 196.1 and 483.4 GPa, respectively. These results indicate that the interstitial hydrogen atom induces a distinct decrease in stiffness. This distinct decrease caused by interstitial hydrogen was also observed in nanoindentation experiments for TWIP steels [53,54]. For Al-containing austenite, the calculated bulk, shear, and Young’s moduli are 296.8, 180.5, and 450.1 GPa, respectively. When interstitial hydrogen atom occupies the octahedral interstitial site, the bulk, shear, and Young’s moduli are 293.1, 181.7, and 451.8 GPa, respectively. Compared with that in Al-free austenite, the change in elastic moduli of Al-containing austenite is negligible, indicative of effective suppression of stiffness deterioration. This phenomenon was also confirmed by experimental observation in previous work [54]. These results about elastic constants demonstrated that Al addition effectively retarded the decrease in the stiffness of austenite steel. The possible reason for this is that interstitial hydrogen atoms can be well accommodated by Al-containing austenite which has a larger unit cell size and better hydrogen-trap capability, thus causing relatively low compressive stress in austenite and a slight decrease in the elastic moduli of steel material.

## 4. Conclusions

In summary, occupying site preference, binding energies and diffusion barrier of interstitial hydrogen atoms in austenites, lattice compressive stress caused by interstitial hydrogen atoms, and elastic constants of bulk austenite steel were theoretically studied at the GGA-PBE/USP level of theory to understand the HE mechanism and the improved resistance of Al-containing austenite to HE. 

Our results demonstrate that hydrogen atoms show interstitial site preference both in Al-free austenite and in Al-containing austenite. In Al-containing austenite interstitial hydrogen atoms have higher binding energies and a diffusion barrier, indicating that Al addition favors a decrease in hydrogen mobility and limits hydrogen diffusion in austenite steel. In both Al-free austenite and Al-containing austenite, interstitial hydrogen atoms have induced a remarkable increase in lattice compressive stress, but the influence in Al-containing austenite is distinctly slighter than that in Al-free austenite. The calculated elastic constants reveal that interstitial hydrogen atoms induce a distinct decrease in bulk, shear, and Young’s moduli of Al-free austenite, whereas it does not show remarkable influence on the Al-containing austenite, this demonstrates that the hydrogen embrittlement effect has been remarkably alleviated by the presence of Al atom in austenitic steel.

## Figures and Tables

**Table 1:** The preferential occupying site of interstitial hydrogen atoms in Al-free and Al-containing austenites and the binding energies (eV) between austenites and interstitial hydrogen atoms.

Magnetic State	Al-Free Austenite		Al-Containing Austenite	
	NM State	FM State	NM State	FM State
1 H interstitial complex	O site 3.24 eV	T site 2.44 eV	O’ site 3.85 eV	O’ site 2.99 eV
2 H interstitial complex	O and T site 5.48 eV	both at T site 5.36 eV	O and O’ site 6.81 eV	both at T site 5.50 eV

**Table 2:** The lattice parameters and volume of Fe_4_ and Fe_3_Al_1_ nit cells.

		Fe_4_	Fe_4_H	Fe_3_Al_1_	Fe_3_Al_1_H	Fe_4_@Fe_108_	Fe_4_H@Fe_108_	Fe_3_Al_1_@Fe_106_Al_2_	Fe_3_Al_1_H@Fe_106_Al_2_
Lattice Vectors (Å)	a	3.446	3.502	3.506	3.585	3.437	3.458	3.435	3.553
	b	3.446	3.502	3.504	3.587	3.434	3.458	3.549	3.554
	c	3.446	3.502	3.604	3.590	3.454	3.458	3.550	3.523
Cell volume (Å^3^)		40.92	42.95	44.29	46.17	40.77	41.35	43.28	44.48

**Table 3:** The calculated elastic constants *C*_ij_, bulk modulus *B*, shear modulus *G*, Young’s modulus *E* (in GPa) of Fe_108_, Fe_108_H, Fe_106_Al_2,_ and Fe_106_Al_2_H in NM state.

	Elastic Constants *C*_ij_ (GPa)			Bulk Modulus *B* (GPa)	Shear Modulus *G* (GPa)			Young’s Modulus *E* (GPa)
	*C*_11_	*C*_12_	*C*_44_		*G_V_*	*G_R_*	*G*	
Fe_108_	473.9	230.1	285.6	311.3	220.1	185.8	203.0	500.2
Fe_108_H	454.0	224.7	281.0	301.2	214.4	177.8	196.1	483.4
Fe_106_Al_2_	440.3	225.0	255.1	296.8	196.1	164.8	180.5	450.1
Fe_106_Al_2_H	433.7	222.8	261.6	293.1	199.1	164.3	181.7	451.8

## Appendix A

The structures of interstitial compounds containing two hydrogen and relative energy.

## References

[1] Alexopoulos N.D., Velonaki Z., Stergiou C.I., Kourkoulis S.K. (2016). The effect of artificial ageing heat treatments on the corrosion-induced hydrogen embrittlement of 2024 (Al-Cu) aluminium alloy. Corros. Sci..

[2] Pouillier E., Gourgues A.F., Tanguy D., Busso E.P. (2012). A study of intergranular fracture in an aluminium alloy due to hydrogen embrittlement. Int. J. Plast..

[3] Koyama M., Akiyama E., Tsuzaki K. (2012). Hydrogen embritllement in a Fe-Mn-C ternary twinning-induced plasticity steel. Corros. Sci..

[4] Seita M., Hanson J.P., Gradecak S., Demkowicz M.J. (2015). The dual role of coherent twin boundaries in hydrogen embrittlement. Nat. Commun..

[5] Dadfarnia M., Nagao A., Wang S., Martin M.L., Somerday B.P., Sofronis P. (2015). Recent advances on hydrogen embrittlement of structural materials. Int. J. Fract..

[6] Mittal S.C., Prasad R.C., Deshmukh M.B. (1994). Effect of hydrogen on fracture of austenitic Fe-Mn-Al steel. ISIJ Int..

[7] So K.H., Kim J.S., Chun Y.S., Park K.T., Lee Y.K., Lee C.S. (2009). Hydrogen delayed fracture properties and internal hydrogen behavior of a Fe-18Mn-1.5Al-0.6C TWIP steel. ISIJ Int..

[8] Ronevich J.A., Speer J.G., Matlock D.K. (2010). Hydrogen embrittlement of commercially produced advanced high strength sheet steels. SAE Int. J. Mater. Manuf..

[9] Gutierrez-Urrutia I., Raabe D. (2011). Dislocation and twin substructure evolution during strain hardening of an Fe-22 wt.% Mn-0.6 wt.% C TWIP steel observed by electron channeling contrast imaging. Acta Mater..

[10] Koyama M., Akiyama E., Tsuzaki K., Raabe D. (2013). Hydrogen-assisted failure in a twinning-induced plasticity steel studied under in situ hydrogen charging by electron channeling contrast imaging. Acta Mater..

[11] Troiano A.R. (2016). The role of hydrogen and other interstitials in the mechanical behavior of metals. Metall. Microstruct. Anal..

[12] Beachem C.D. (1972). A new model for hydrogen-assisted cracking (hydrogen embrittlement). Metall. Mater. Trans. B.

[13] Birnbaum H.K., Sofronis P. (1994). Hydrogen-enhanced localized plasticity-a mechanism for hydrogen-related fracture. Mater. Sci. Eng. A.

[14] Abraham D.P., Altstetter C.J. (1995). Hydrogen-enhanced localization of plasticity in an austenitic stainless-steel. Metall. Mater. Trans. A.

[15] Abraham D.P., Altstetter C.J. (1995). The effect of hydrogen on the yield and flow-stress of an austenitic stainless-steel. Metall. Mater. Trans. A.

[16] Asano S., Otsuka R. (1976). The lattice hardening due to dissolved hydrogen in iron and steel. Scr. Metall..

[17] Song J., Curtin W.A. (2013). Atomic mechanism and prediction of hydrogen embrittlement in iron. Nat. Mater..

[18] Park I.J., Lee S.M., Jeon H.H., Lee Y.K. (2015). The advantage of grain refinement in the hydrogen embrittlement of Fe-18Mn-0.6C twinning-induced plasticity steel. Corros. Sci..

[19] Kim K.H., Park H.C., Lee J., Cho E., Lee S.M. (2013). Vanadium alloy membranes for high hydrogen permeability and suppressed hydrogen embrittlement. Scr. Mater..

[20] Suzuki A., Yukawa H., Nambu T., Matsumoto Y., Murata Y. (2016). Analysis of pressure-composition-isotherms for design of non-Pd-based alloy membranes with high hydrogen permeability and strong resistance to hydrogen embrittlement. J. Membr. Sci..

[21] Park I.J., Jo S.Y., Kang M., Lee S.M., Lee Y.K. (2014). The effect of Ti precipitates on hydrogen embrittlement of Fe-18Mn-0.6C-2Al-xTi twinning-induced plasticity steel. Corros. Sci..

[22] Zamanzade M., Vehoff H., Barnoush A. (2014). Cr effect on hydrogen embrittlement of Fe3Al-based iron aluminide intermetallics, surface or bulk effect. Acta Mater..

[23] Lee S.M., Park I.J., Jung J.G., Lee Y.K. (2016). The effect of Si on hydrogen embrittlement of Fe-18Mn-0.6C-xSi twinning-induced plasticity steels. Acta Mater..

[24] Chun Y.S., Park K.T., Lee C.S. (2012). Delayed static failure of twinning-induced plasticity steels. Scr. Mater..

[25] Park K.T., Jin K.G., Han S.H., Hwang S.W., Choi K., Lee C.S. (2010). Stacking fault energy and plastic deformation of fully austenitic high manganese steels, effect of Al addition. Mater. Sci. Eng. A.

[26] Chin K.G., Kang C.Y., Shin S.Y., Hong S., Lee S., Kim H.S., Kim K.H., Kim N.J. (2011). Effects of Al addition on deformation and fracture mechanisms in two high manganese TWIP steels. Mater. Sci. Eng. A.

[27] Song S.W., Kwon Y.J., Lee T., Lee C.S. (2016). Effect of Al addition on low-cycle fatigue properties of hydrogen-charged high-Mn TWIP steel. Mater. Sci. Eng. A.

[28] Dieudonné T., Marchetti L., Wery M., Jomard F., Miserque F., Tabarant M., Chêne J., Allely C., Cugy P., Scott C.P. (2014). Role of copper and aluminum on the corrosion behavior of austenitic Fe–Mn–C TWIP steels in aqueous solutions and the related hydrogen absorption. Corros. Sci..

[29] Dieudonné T., Marchetti L., Wery M., Chêne J., Allely C., Cugy P., Scott C.P. (2014). Role of copper and aluminum additions on the hydrogen embrittlement susceptibility of austenitic Fe-Mn-C TWIP steels. Corros. Sci..

[30] Ryu J.H., Kim S.K., Lee C.S., Suh D.W., Bhadeshia H.K.D.H. (2013). Effect of aluminum on hydrogen-induced fracture behavior in austenitic Fe-Mn-C steel. Proc. R. Soc. A Math. Phys. Eng. Sci..

[31] Song E.J., Bhadeshia H.K.D.H., Suh D.W. (2014). Interaction of aluminium with hydrogen in twinning-induced plasticity steel. Scr. Mater..

[32] Segall M.D., Lindan P.J.D., Probert M.J., Pickard C.J., Hasnip P.J., Clark S.J., Payne M.C. (2002). First-Principles Simulation, Ideas, Illustrations and the CASTEP Code. J. Phys. Condens. Matter.

[33] Perdew J.P., Burke K., Ernzerhof M. (1996). Generalized Gradient Approximation Made Simple. Phys. Rev. Lett..

[34] Vanderbilt D. (1990). Soft Self-consistent Pseudopotentials in a Generalized Eigenvalue Formalism. Phys. Rev. B.

[35] Pfrommer B.G., Cote M., Louie S.G., Cohen M.L. (1997). Relaxation of Crystals with the Quasi-Newton Method. J. Comput. Phys..

[36] Govind N., Petersen M., Fitzgerald G., King-Smith D., Andzelm J.A. (2003). Generalized Synchronous Transit Method for Transition State Location. Comput. Mater. Sci..

[37] Henkelman G., Jónsson H. (2000). Improved Tangent Estimate in the Nudged Elastic Band Method for Finding Minimum Energy Paths and Saddle Points. J. Chem. Phys..

[38] Abbasi A., Dick A., Hickel T., Neugebauer J. (2011). First-principles investigation of the effect of carbon on the stacking fault energy of Fe-C alloys. Acta Mater..

[39] Korzhavyi P.A., Abrikosov I.A., Johansson B. (1999). First-principles calculations of the vacancy formation energy in transition and noble metals. Phys. Rev. B.

[40] Herper H.C., Hoffmann E., Entel P. (1999). Ab initio full-potential stduy of the structural and magnetic pahse stability of iron. Phys. Rev. B.

[41] Jiang D.E., Carter E.A. (2003). Carbon dissolution and diffusion in ferrite and austenite from first principles. Phys. Rev. B.

[42] Nazarov R., Hickel T., Neugebauer J. (2010). First-principles study of the thermodynamics of hydrogen-vacancy interaction in fcc iron. Phys. Rev. B.

[43] Klaver T.P.C., Hepburn D.J., Ackland G.J. (2012). Defect and solute properties in dilute Fe-Cr-Ni austenitic alloys from first principles. Phys. Rev. B.

[44] Wang S.Q., Ye H.Q. (2003). Ab initio elastic constants for the lonsdaleite phase of C, Si and Ge. J. Phys. Condens. Matter.

[45] Mouhat F., Coudert F.-X. (2014). Necessary and sufficient elastic stability conditions in various crystal systems. Phys. Rev. B.

[46] Lu L., Zhang S., Zhang H., Li F., Liang F., Li Y. (2016). Structures and mechanical properties of Fe- and Cr-incorporated β-Si_5_AlON_7_, First-principles study. Ceram. Int..

[47] Teus S.M., Shivanyuk V.N., Shanina B.D., Gavriljuk V.G. (2007). Effect of hydrogen on electronic structure of fcc iron in relation to hydrogen embrittlement of austenitic steels. Phys. Stat. Sol. A.

[48] Migliori A., Sarrao J.L., Visscher W.M., Bell T.M., Lei M., Fisk Z., Leisure R.G. (1993). Resonant ultrasound spectroscopic techniques for measurement of the elastic moduli of solids. Phys. B Condens. Matter.

[49] Reeh S., Music D., Gebhardt T., Kasprzak M., Jäpel T., Zaefferer S., Raabe D., Richter S., Schwedt A., Mayer J. (2012). Elastic properties of face-centred cubic Fe-Mn-C studied by nanoindentation and ab initio calculations. Acta Mater..

[50] Sevillano J.G. (2009). An alternative model for the strain hardening of FCC alloys that twin, validated for twinning-induced plasticity steel. Scr. Mater..

[51] Guo G.Y., Wang H.H. (2000). Gradient-Corrected Density Functional Calculation of Elastic Constants of Fe, Co and Ni in bcc, fcc and hcp Structures. Chin. J. Phys..

[52] Asker C., Vitos L., Abrikosov I.A. (2009). Elastic constants and anisotropy in FeNi alloys at high pressures from first-principles calculations. Phys. Rev. B.

[53] Nibur K.A., Bahr D.F., Somerday B.P. (2006). Hydrogen effects on dislocation activity in austenitic stainless steel. Acta Mater..

[54] Han D.K., Kim Y.M., Han H.N., Bhadeshia H.K.D.H., Suh D.-W. (2014). Hydrogen and aluminium in high-manganese twinning-induced plasticity steel. Scr. Mater..

